# Supplementary material for: Transient Elastography as the First-Line Assessment of Liver Fibrosis and Its Correlation with Serum Markers
Source: Medicina (Kaunas). 2023 Apr 12;59(4):752. doi: 10.3390/medicina59040752 (PMC10146833; doi:10.3390/medicina59040752)
Supplement: Supplementary file 1 [file medicina-59-00752-s001.zip › medicina-2205092-supplementary.pdf]

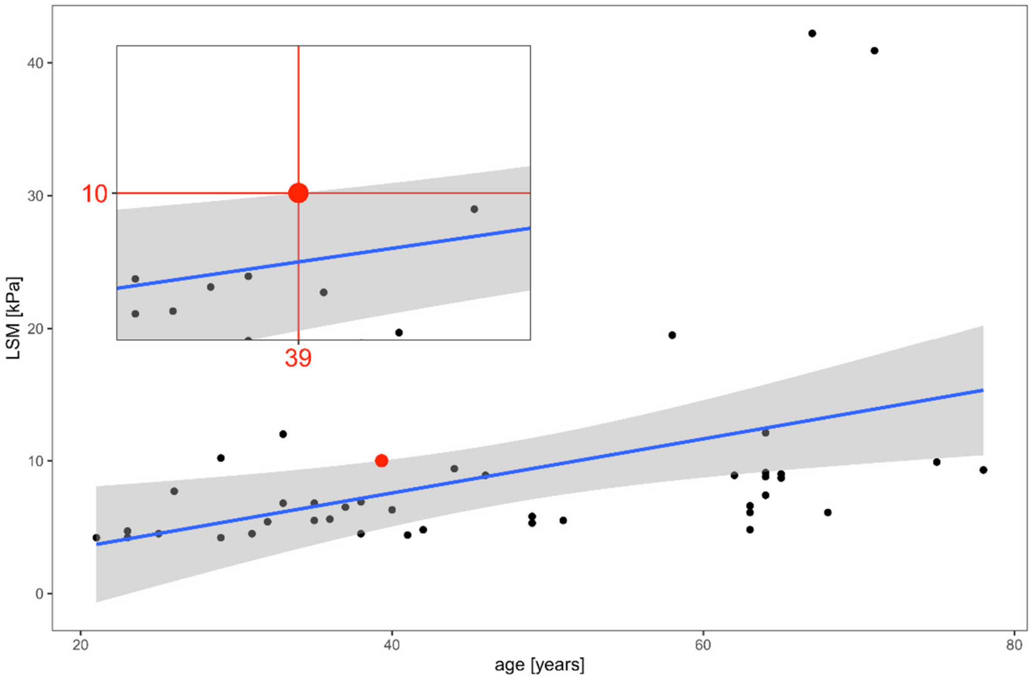

Figure S1. Correlation of LSM and age in patients with viral hepatitis. Advanced liver fibrosis is unlikely in individuals younger than 39.0 years. The blue line represents the linear regression model of the correlation between LSM and age. The black points represent the individual patients. The red point represents the cut-off value with the 95% certainty that individuals younger than 39 years do not have LSM > 10 kPa. The confidence intervals are in grey. LSM, Liver Stiffness Measurement.

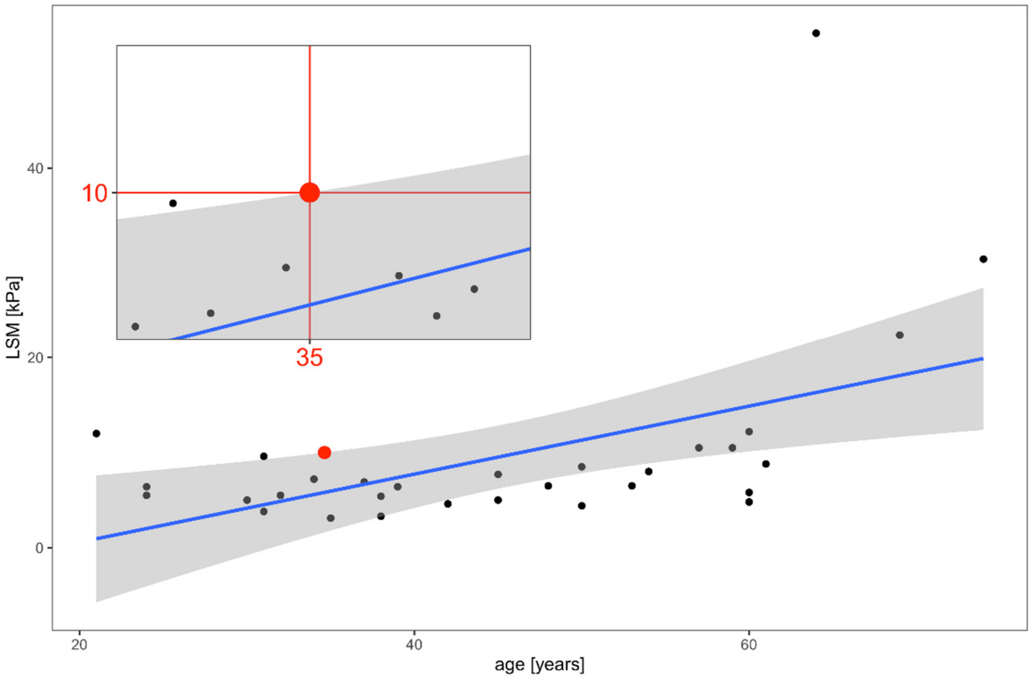

Figure S2. Correlation of LSM and age in patients with NAFLD/NASH. Advanced liver fibrosis is unlikely in individuals younger than 35.0 years. The blue line represents the linear regression model of the correlation between LSM and age. The black points represent the individual patients. The red point represents the cut-off value with the 95% certainty that individuals younger than 35 years do not have LSM > 10 kPa. The confidence intervals are in grey. LSM, Liver Stiffness Measurement.
